# Using Drift Diffusion and RL Models to Disentangle Effects of Depression On Decision-Making vs. Learning in the Probabilistic Reward Task

**DOI:** 10.5334/cpsy.108

**Published:** 2024-05-03

**Authors:** Daniel G. Dillon, Emily L. Belleau, Julianne Origlio, Madison McKee, Aava Jahan, Ashley Meyer, Min Kang Souther, Devon Brunner, Manuel Kuhn, Yuen Siang Ang, Cristina Cusin, Maurizio Fava, Diego A. Pizzagalli

**Affiliations:** 1 Center for Depression, Anxiety and Stress Research, McLean Hospital, Belmont MA, USA; 2 Harvard Medical School, Boston MA, USA; 3 Depression Clinical and Research Program, Massachusetts General Hospital, Boston MA, USA

**Keywords:** computational modeling, depression, reward, decision-making, anhedonia, placebo

## Abstract

The Probabilistic Reward Task (PRT) is widely used to investigate the impact of Major Depressive Disorder (MDD) on reinforcement learning (RL), and recent studies have used it to provide insight into decision-making mechanisms affected by MDD. The current project used PRT data from unmedicated, treatment-seeking adults with MDD to extend these efforts by: (1) providing a more detailed analysis of standard PRT metrics—response bias and discriminability—to better understand how the task is performed; (2) analyzing the data with two computational models and providing psychometric analyses of both; and (3) determining whether response bias, discriminability, or model parameters predicted responses to treatment with placebo or the atypical antidepressant bupropion. Analysis of standard metrics replicated recent work by demonstrating a dependency between response bias and response time (RT), and by showing that reward totals in the PRT are governed by discriminability. Behavior was well-captured by the Hierarchical Drift Diffusion Model (HDDM), which models decision-making processes; the HDDM showed excellent internal consistency and acceptable retest reliability. A separate “belief” model reproduced the evolution of response bias over time better than the HDDM, but its psychometric properties were weaker. Finally, the predictive utility of the PRT was limited by small samples; nevertheless, depressed adults who responded to bupropion showed larger pre-treatment starting point biases in the HDDM than non-responders, indicating greater sensitivity to the PRT’s asymmetric reinforcement contingencies. Together, these findings enhance our understanding of reward and decision-making mechanisms that are implicated in MDD and probed by the PRT.

Research on the impact of depression on reward processing and reinforcement learning (RL) has flourished (Brown et al., 2021; Dombrovski et al., 2019; Proudfit, 2015) with the development of reward tasks that are sensitive to depressive illness (Halahakoon et al., 2020), improved understanding of brain reward systems (Haber & Knutson, 2010), and increased use of computational models (Gershman et al., 2015; Huys et al., 2016; Jaskir & Frank, 2023). The current study addresses three topics important to the continued success of this work: the complexity of behavior, psychometrics, and the predictive power of reward paradigms. We investigated these topics using the probabilistic reward task (PRT; Pizzagalli et al., 2005). The PRT is commonly used to assess RL in depression, but recent data show that it can also provide insight into decision-making (Dillon et al., 2022; Grange, 2022; Lawlor et al., 2020).

## Behavioral Complexity

**Figure 1:**
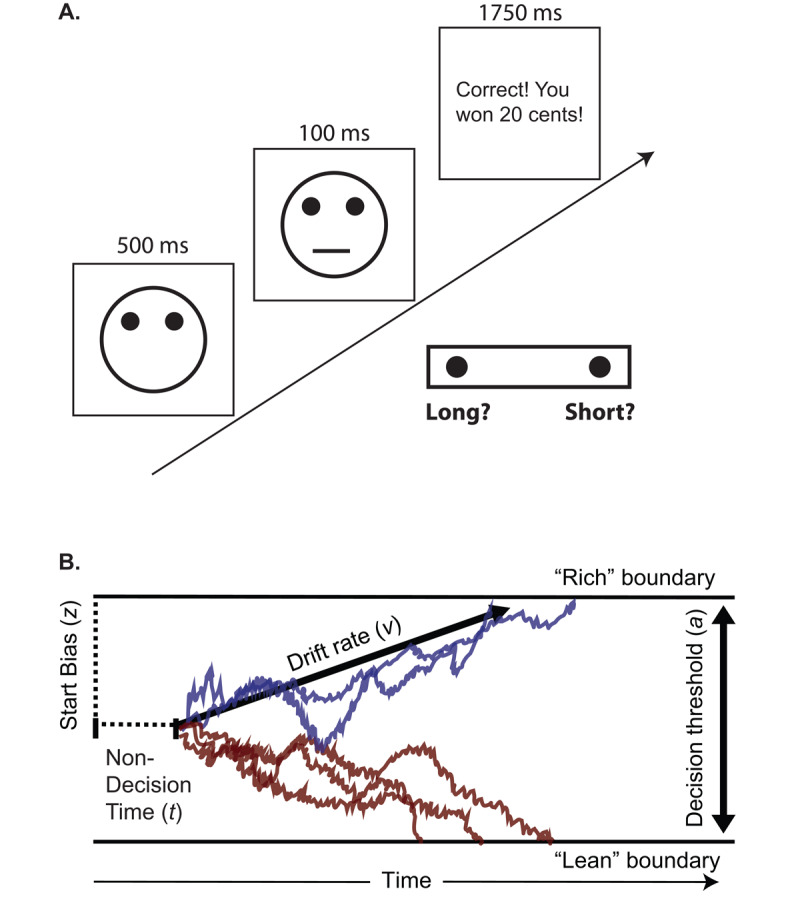
The **(A)** Probabilistic Reward Task (PRT) and **(B)** Drift Diffusion Model. *Note*. (A) PRT trial stucture. (B) Blue and red lines show the drift process reaching the rich and lean boundaries, respectively. The HDDM, as setup in this study, returns one average drift rate per participant.

Behavior in RL tasks can be surprisingly complex. In the PRT (Figure 1A), which is rooted in the signal detection framework (Macmillan & Creelman, 2004), participants must distinguish between two stimuli (schematic mouths) of similar length, and correct identifications of one (rich) stimulus are rewarded three times more often than correct identifications of the other (lean) stimulus. Consequently, participants typically develop a response bias: they respond “rich” more than “lean” (note: we use quotes when referring to “rich” and “lean” responses, but omit them when referring to rich and lean stimuli). Weak response bias may be a marker of anhedonia (Pizzagalli et al., 2005) and many data are consistent with this proposal (Liu et al., 2016; Pizzagalli et al., 2008b,c; Vrieze et al., 2013). Moreover, research has probed neural mechanisms that support response biases (Iturra-Mena et al., 2023; Pizzagalli et al., 2008a; Santesso et al., 2008). This body of work is sufficiently extensive that the PRT appears in the NIH RDoC Matrix as a validated test of reward learning. Nevertheless, in a recent study comparing unmedicated adults with Major Depressive Disorder (MDD) to healthy controls, we identified two aspects of PRT performance that were previously unrecognized (Lawlor et al., 2020).

First, we found that the response bias effect was not uniform: it was strong when response times (RTs) were fast (≤ 0.3 quantile), but weaker when RTs were slower. This indicates that the mechanism(s) underlying response bias likely involve preparation of fast motor actions rather than, for example, sustained stimulus evaluation (White & Poldrack, 2014). Second, reward totals were better predicted by discriminability than by response bias. In other words, the participants who earned the most rewards were those who responded most accurately, not those who developed the strongest tendency to respond “rich”. Given the emphasis on response bias in the PRT literature, this finding was striking. Moreover, it had a consequence: although no group difference in response bias emerged, controls earned significantly more rewards than depressed adults due to better discriminability. This indicates that weak response bias may not necessarily indicate anhedonia: by responding accurately, a participant with excellent discriminability could form no response bias and yet harvest more rewards, more quickly, than a participant with a stronger response bias but poorer discriminability. We replicated these results in a study of social anxiety disorder (SAD; Dillon et al., 2022), although here the socially anxious adults showed better discriminability (and earned more rewards) than healthy controls, especially after gaze training (Lazarov et al., 2017) for their anxiety. In the current study, we attempted to replicate our findings in a new depressed sample.

## Models and Psychometrics

The results just described suggest that there are multiple ways to model the PRT, with implications for how to conceptualize task performance and MDD pathophysiology. Past research (Huys et al., 2013) successfully accounted for PRT data using an RL model, called the “belief” model, that envisions participants updating the estimated value of the rich and lean stimuli by computing trial-level prediction errors (PEs). This is sensible given the emphasis on rewards in the PRT, strong relationships between PEs and dopamine (Schultz, 1998), and the long-standing hypothesis that depression involves dopamine dysfunction (Dunlop & Nemeroff, 2007; Nestler & Carlezon, 2006; Treadway & Zald, 2011). The belief model has parameters for reward sensitivity and learning rate, two important contributors to RL, and a meta-analysis found relationships between MDD, anhedonia, and reward responsivity (Huys et al., 2013). In short, focusing on RL has been productive.

In the PRT, however, the reward probabilities for correctly identifying the rich and lean stimuli are fixed and reward magnitude does not change. Consequently, trial-level value updates may not be critical. Furthermore, the reward probability gap across the response options is so large that precise value estimates may not be necessary—a simple policy (press “rich”) based on rough approximations may suffice (Jaskir & Frank, 2023). Finally, participants are asked to make a difficult perceptual decision on each trial. This is unusual in the RL space, but tasks with similar attributes have been successfully analyzed with sequential sampling models, including the drift diffusion model (DDM; Ratcliff, 1978; Ratcliff & McKoon, 2008), for decades (Newsome et al., 1989; Platt & Glimcher, 1999; Sugrue et al., 2004). Inspired by these examples, in our prior study of MDD (Lawlor et al., 2020) we analyzed the PRT data with the Hierarchical Drift Diffusion Model (HDDM; Wiecki et al., 2013).

The DDM conceptualizes decision-making as a process of evidence accumulation to thresholds (Figure 1B). When fit to the data in Lawlor et al. (2020), it captured the novel results mentioned earlier. Specifically, the DDM modeled the dependency between response bias and RT by moving the starting point of the diffusion process closer to the rich boundary, such that relatively little evidence needed to accumulate to elicit a “rich” response. Poorer discriminability in the MDD group was accounted for by reducing the drift rate, which captures the speed of evidence accumulation. Drift rate strongly predicted discriminability, supporting the conclusion that slow drift rate in MDD led to poor discriminability, which led to low reward totals. In short, the DDM—although not designed to address RL—provided an excellent account of the data. The relationships among these parameters replicated in our study of SAD (Dillon et al., 2022). Attempting to replicate these relationships again, and to confirm that drift rate is slow in adults with MDD, were aims of the current analysis.

Therefore, we fit our data with the HDDM and the RL models in Huys et al (2013), including the belief model. We used simulations to assess each model’s ability to capture behavior, and we investigated two psychometric properties: internal consistency and retest reliability. Internal consistency indexes a variable’s within-session stability and serves as a floor for the strength of all other relationships—a measure cannot be more strongly related to another measure than it is to itself (Infantolino et al., 2018; Luking et al., 2017). We also assessed retest reliability. In this study, data were collected twice about three weeks apart (but see below for the limitation of an intervening manipulation), allowing us to examine the reproducibility of parameter estimates. In our prior work (Dillon et al., 2022; Lawlor et al., 2020), HDDM parameters estimated from PRT data showed good to excellent internal consistency. Our data in SAD (Dillon et al., 2022), and larger datasets from different tasks (Lerche & Voss, 2017; Schubert et al., 2016; Yap et al., 2012), also indicate that the retest reliability of DDM parameters is adequate to good. By contrast, the psychometrics of the belief model have not been studied. Examining psychometrics is important for precision medicine: measures with high internal consistency are needed to predict outcomes for individuals. As described next, predicting treatment responses was the goal of the larger study for which the current data were collected.

## Predicting Placebo Responses

The data described here come from a project examining whether reward tasks can predict placebo responses. Such predictions could facilitate placebo responding in clinical settings (to reap therapeutic benefits) while removing placebo responders from drug trials (to more easily detect drug effects). The primary hypothesis was that individuals with better reward system function would be more likely to show placebo responses (Brown & Peciña, 2019; Peciña et al., 2021).

To test this account, adults with MDD completed a positron emission tomography (PET)/magnetic resonance imaging (MRI) scan to assess mesolimbic dopaminergic reward system activity before beginning a clinical trial in which they were initially randomized to placebo or to the atypical antidepressant bupropion, which is a dopamine/norepinephrine reuptake inhibitor (Learned-Coughlin et al., 2003); most participants who received placebo and did not respond were switched to bupropion (see Methods for details; see Figure S1 for a summary). About three weeks later, participants completed the tasks a second time. Details of the trial and the PET/MRI data will be presented elsewhere. Here we focus on PRT data, collected from a subset of participants after the PET/MRI scans. We used the data to address two questions. First, do pre-treatment model parameters or standard PRT metrics (response bias, discriminability) predict placebo responses? Second, do pre-treatment scores on these variables predict responses to bupropion in placebo non-responders? As detailed below, low statistical power limited our ability to answer these questions. Nonetheless, the data may provide leads for future investigations.

## Summary

This PRT analysis had four aims: (a) replicate the dependency between response bias and RT, and the finding that reward totals are better predicted by discriminability vs. response bias; (b) fit the data with the HDDM and the belief model, with the expectation that these models will provide insight into mechanisms underlying discriminability and response bias, and will uncover slow drift rates in this depressed sample; (c) examine internal consistency and retest reliability of model parameters; and (d) determine whether response bias, discriminability, or any model parameter can predict responses to placebo or responses to bupropion in placebo non-responders.

## Methods

Data and code are available at the Open Science Framework (OSF) at https://osf.io/347rm. The study was not pre-registered.

### Participants and Protocol

This research was performed following a protocol approved by the Mass General Brigham Institutional Review Board (“Neurobiological underpinnings of placebo response in depression”, #2014P000889), and written informed consent was obtained. Prospective participants were recruited by the Depression and Clinical Research Program at Massachusetts General Hospital (MGH). Inclusion criteria included: (a) meeting Diagnostic and Statistical Manual IV (DSM-IV; First et al., 2002) criteria for MDD; (b) 18–45 years old; (c) score of 17 or greater on the Hamilton Depression Rating Scale-31 (HAMD-31; Williams et al., 1988); (d) continuing to meet criteria for current MDD and Clinical Global Impression (CGI; Guy, 1976) improvement scores ≤ 3 in the interval between the screen and Session 1 visit; and (e) no failed antidepressant trials of adequate dose and duration, as defined by the MGH Antidepressant Treatment History Questionnaire (ATHQ; Chandler et al., 2010). Exclusion criteria minimized comorbidity and eliminated risks associated with neuroimaging; see the Supplement for details.

Eligible individuals were enrolled in a clinical trial. The trial and clinical data will be detailed elsewhere; because the current manuscript is focused on the PRT, a summary is given. Briefly, the trial used a sequential parallel comparison design (Fava et al., 2003) to increase the number of placebo responders. Participants were randomized to placebo or 300 mg bupropion in a 7:1 ratio (87.5% placebo, 12.5% bupropion) and were told that bupropion is a “fast-acting antidepressant” because expectations of success can drive placebo responses (Zubieta & Stohler, 2009) and affect reward circuitry (Lidstone et al., 2010). After four weeks, participants randomized to bupropion stayed on bupropion, whereas placebo responders and non-responders were re-randomized to placebo or bupropion in a 1:7 ratio (12.5% placebo vs. 87.5% bupropion, computed separately for placebo responders and non-responders). Depression was assessed throughout with the HAMD-31. Responder status was assessed four weeks post-baseline and defined as a 50% or greater reduction in HAMD-31 score; drop outs were considered non-responders.

PRT data were collected before randomization and again three weeks later; these timepoints are referred to as Sessions 1 and 2. Because placebo responses often develop in about two weeks (Posternak & Zimmerman, 2005; Quitkin et al., 1991), scheduling Session 2 three weeks after Session 1 should assess those responses. Note that while treatment continued after Session 2, we call this session “post-treatment” because it occurred after randomization.

### PRT

PRT methods followed prior reports (Pizzagalli et al., 2005). Participants sat at a PC with their index fingers on the “v” and “m” keys of a keyboard and completed three blocks of 100 trials. As shown in Figure 1A, trials began with presentation of a schematic face (500 ms) onto which a “short” (11.5 mm) or “long” (13.0 mm) mouth was flashed (100 ms). The task was to indicate, as quickly as possible, which length was shown. Most trials yielded no feedback, but 20 cent rewards were delivered three times more often for correct identifications of one mouth (the rich stimulus, up to 30 rewards) vs. the other (the lean stimulus, up to 10 rewards). Assignment of lengths to rich/lean conditions was counterbalanced across participants, and the keys used for “rich” vs. “lean” responses were counterbalanced across sessions. Participants were paid $15.80 or $16.20.

### Quality Control

*A prior* quality control assessments were used to exclude poor quality datasets; these and all subsequent analyses were conducted using Python in Jupyter notebooks (Kluyver et al., 2016). Outliers were defined as trials with raw RTs faster than 150 ms or slower than 2,500 ms, or where the ln(RT) exceeded the participant’s mean ln(RT) ± 3S.D., computed separately for rich vs. lean trials. Blocks were QC failures if there were: fewer than 80 non-outlier trials; a rich/lean reward ratio < 2.0; fewer than 20 rewarded rich trials; or fewer than 6 rewarded lean trials. If any blocks was marked as a QC failure, the dataset was excluded.

### Signal Detection Analyses

Response bias and discriminability were computed by counting the number of correct/incorrect rich and lean responses per block and entering them in these formulas (Pizzagalli et al., 2005):


\[
response\;bias = 0.5*lo{g_{10}}(\frac{{ric{h_{correct}}*lea{n_{incorrect}}}}{{ric{h_{incorrect}}*lea{n_{correct}}}})
\]

\[
discriminability = 0.5*lo{g_{10}}(\frac{{ric{h_{correct}}*lea{n_{correct}}}}{{ric{h_{incorrect}}*lea{n_{incorrect}}}})
\]

Counts were initialized at 0.5 to avoid division by zero (Hautus, 1995).

### Relationships Among Behavioral Variables

We studied relationships among PRT variables to better understand how the task is performed, to identify regularities that might be captured by the models, and to replicate prior findings (Dillon et al., 2022; Lawlor et al., 2020). First, quantile-probability plots were used to examine the RT distribution. Based on earlier work, we expected a strong right skew in our depressed sample. In other words, we anticipated that many trials would be characterized by slow RTs, leading to a long right tail when the RT distribution is plotted horizontally. This is important because the shape of the RT distribution yields predictions for the HDDM: a strongly right-skewed distribution is often well-fit by a slow drift-rate (Ratcliff, 1978). Second, the quantile-probability plots were used to determine whether the rich > lean accuracy effect, which captures response bias, varied by RT. We expected the rich > lean accuracy difference to be larger for faster vs. slower RTs. Finally, we again expected reward totals to be more strongly related to discriminability than response bias, because accurate responding allows participants to efficiently collect rewards on rich and lean trials.

### HDDM

We fit the HDDM (version 0.7.5) to trial-level choice and accuracy data, separately by session. We retained the default priors (Wiecki et al., 2013) and modeled the data using the HDDM’s *StimulusCoding* tool. The model was the same as in prior studies (Dillon et al., 2022; Lawlor et al., 2020):

m = hddm.StimCoding(data, include = ‘z’, stim_col = ‘stim’, split_param = ‘v’)

This approach estimates the starting point bias (z) and returns an absolute value for drift rate (v), which is coded –v and +v for trials in which the lower (0 = “lean” response) vs. upper (1 = “rich” response) boundary is reached.

Models were estimated by drawing three chains of 2,500 samples from the posterior (burn-in: 500 samples). Chains were concatenated and convergence was assessed by inspecting posterior distributions and by checking that the maximum \[
\hat R
\] did not exceed 1.1 (Gelman & Rubin, 1992) (max \[
\hat R
\] : Session 1 = 1.003; Session 2 = 1.016). Next, the HDDM’s *post_pred_gen* tool was used to generate 500 simulated datasets per participant, and *post_pred_stats* was used to check if simulated accuracy and RT matched the observed data.

#### Relationship with standard metrics

To facilitate interpretation, we regressed each participant’s (across-block) mean response bias and discriminability against the HDDM parameters. We expected response bias to be most strongly related to starting point bias and discriminability to be most strongly related to drift rate and threshold (because response accuracy is facilitated by rapid evidence accumulation and wide boundaries, which maximize the likelihood of the drift process reaching the correct boundary).

#### RLDDM

The data were also fit with the Reinforcement Learning Drift Diffusion Model (RLDDM; Pedersen & Frank, 2020). Briefly, the RLDDM uses trial-level PEs to assign values to (stimulus, action) pairs—e.g., “rich” responses to rich stimuli—in a Q-learning framework, but it uses the DDM (rather than the softmax function) as the choice rule: here, drift rate is scaled on each trial by the difference in Q-values for the two response options. It was unclear if the combination of RL plus the DDM would improve on the HDDM fits. To investigate this, we used Deviance Information Criterion (DIC) values and posterior predictive checks to compare RLDDM vs. HDDM fits.

### RL Models

The data were next fit with RL models successfully used in prior PRT research (Huys et al., 2013; code for all models at https://github.com/mpc-ucl/emfit). Group priors were computed via expectation-maximization (Do and Batzoglou, 2008) and Laplace approximation of posterior distributions was used to compute subject-specific parameters. To compare model fit, integrated group-level Bayesian Information Criterion (iBIC) was used. Consistent with prior findings (Huys et al., 2013), the most parsimonious account of the data was provided by the belief model, so for brevity we do not discuss the other RL models. The belief model postulates that rewards serve to inform two uncertainty-weighted stimulus-action associations (since participants may be unsure about which stimulus was presented in a given trial). The model has five parameters: reward sensitivity, learning rate, belief, instruction sensitivity, and initial action bias.

A detailed treatment of the belief model is in Huys et al. (2013), but here we provide an overview. Briefly, this is a Q-learning model in which prediction errors (PEs)—the discrepancy between expected and actual rewards (0 = no reward, 1 = reward)—serve to update Q-value estimates for each action/stimulus pair (e.g., responding “rich” to the rich stimulus). *Reward sensitivity* scales reward impact: when it is larger, rewards have greater impact, and when it is smaller they are devalued. This allows for the possibility that rewards are “liked” more or less by different participants. *Learning rate* is also a scaling factor—it controls the speed with which PEs affect Q-values. When learning rate is higher, Q-values change more rapidly from trial-to-trial, whereas when it is lower, the Q-values change more gradually. In the belief model, participants estimate four Q-values, one each for the 2 *StimulusType* × 2 *ResponseType* design. The similarity of stimuli, however, makes it hard for participants to know which stimulus was shown on any trial. The *belief* parameter captures this uncertainty. *Instruction sensitivity* tracks accuracy: it covaries with participants’ ability to correctly identify the rich vs. lean stimulus. Finally, *action bias* corresponds to a preference for one of the two responses. To avoid non-Gaussianity, parameters were transformed. To conduct posterior predictive checks, we used the best-fitting parameters to simulate 500 datasets per particpant, as done with the HDDM.

#### Relationship with standard metrics

We regressed response bias and discriminability against the belief model’s five parameters.

### Psychometrics

To examine internal consistency, the data were split into odd and even trials and submitted to signal detection, HDDM, and belief model analyses. The belief model involves value updates driven by PEs on sequential trials, which might make this approach suboptimal. Therefore, we also split each participant’s data in half and examined the consistency of analyses conducted on each half. The Spearman-Brown (SB) formula (Brown, 1910; Spearman, 1910) was calculated to measure internal consistency:


\[
SB = \frac{{2*r}}{{1 + r}}
\]

Here, *r* is the Pearson correlation coefficient for odd vs. even trials, or for the first vs. second half of trials.

To examine test-retest reliability, Pearson correlations comparing Sessions 1 and 2 were computed for mean (across-block) response bias and discriminability values, and for all model parameters; the mean ± SD days between sessions was 24 ± 5. Recall that all participants received either placebo or bupropion between Sessions 1 and 2. Consequently, estimates of test-retest reliability should be interpreted cautiously as they may be affected by treatment.

### Predicting Responses to Placebo and Bupropion

To examine whether pre-treatment PRT data could predict placebo responses, we ran a 2 (*PlaceboResponder:* yes, no) × 3 (*Block*) ANOVA on Session 1 response bias and discriminability. These ANOVAs were repeated using Session 2 PRT data; here the expectation was that Session 2 response bias would be higher in placebo responders vs. non-responders. Next, the HDDM Session 1 and 2 analyses were re-run, this time allowing all parameters to vary by placebo response status. For the belief model, all five parameters were submitted to between-group *t*-tests (placebo responder vs. non-responder) for Sessions 1 and 2.

Finally, we identified placebo non-responders re-randomized to bupropion. Session 1 and 2 data from these participants were analyzed in 2 (*BupropionResponder:* yes, no) × 3 (*Block*) ANOVAs on response bias and discriminability. The HDDM was re-estimated allowing all parameters to vary as a function of bupropion response status, and between-groups *t*-tests (bupropion responders vs. non-responders) were computed for all belief model parameters.

### Statistics

Statistical tests include linear regressions, ANOVAs, pairwise comparisons, and *t*-tests conducted in R, version 4.0.5 “Shake and Throw” (R Core Team, 2023). Packages included *afex* for ANOVAs (Singmann et al., 2016), *emmeans* for pairwise comparisons (Lenth et al., 2020), and *cocor* for comparing correlations (Diedenhofen & Musch, 2015).

## Results

### Sample Characteristics and Responder Status

Session 1 data were collected from 59 participants;10 QC failures were discarded, leaving 49 datasets. Session 2 data were collected from 57 participants; 13 QC failures were discarded, leaving 44 datasets. The two most common problems were losing too many trials to outlier RTs (30% of failures in Session 1, 22% in Session 2) and having too few usable rich trials in which a reward was delivered (33% of failures in Session 1, 41% in Session 2). The elevated rate of QC failures may reflect fatigue as the PRT was completed after the PET/fMRI session.

The samples were predominantly White and not Hispanic or Latino, with a roughly equal mix of males and females and a mean education level equivalent to some years of college (see Table S1). Due to scheduling issues, 34 participants provided usable data from Sessions 1 and 2. Consequently, we provide pre- and post-treatment HAMD scores separately for Session 1 and 2, since these two samples were not entirely overlapping.

Of 49 Session 1 participants, 43 were randomized to placebo: 11 responded, 32 did not. There was no difference in baseline HAMD (mean ± S.D., responders: 29.82 ± 6.08; non-responders: 32.41 ± 6.84), but four weeks later scores were lower in responders (10.45 ± 4.99) vs. non-responders (26.30 ± 7.03), *t*(39) = 6.84, *p* < 0.001. The remaining 6 participants were randomized to bupropion: 3 responded, 3 did not. HAMD scores were similar at baseline (responders: 26.67 ± 10.02; non-responders: 40.67 ± 6.66), but differed four weeks later (responders: 7.00 ± 7.94; non-responders: 37.33 ± 10.21); no test conducted given small samples.

Of the 44 Session 2 participants, 40 had been randomized to placebo: there were 14 responders vs. 26 non-responders (note: there were more placebo responders at Session 2 vs. 1 because not all responders completed the Session 1 PRT, and due to Session 1 QC failures). There was no difference in baseline HAMD scores for placebo responders (29.07 ± 7.60) vs. non-responders (31.19 ± 6.71), but after four weeks scores were lower in responders (10.29 ± 5.43) vs. non-responders (26.35 ± 6.89), *t*(38) = 7.54, *p* < 0.001. The remaining four participants were randomized to bupropion; three responded. In this small group, baseline HAMD scores were similar (responders: 26.00 ± 9.00; non-responder: 33) but four weeks later scores were lower in responders (6.00 ± 6.24); the non-responder’s score (33) did not change.

Finally, Session 1 data were collected from 26 placebo non-responders who were re-randomized to bupropion and subsequently classified as responders (*n* = 12) or non-responders (*n* = 14). HAMD scores did not differ at baseline (responders: 30.25 ± 6.37; non-responders: 34.14 ± 6.59), but they differed at the final clinical session (4 weeks after Session 2: responders: 9.67 ± 4.58; non-responders: 25.82 ± 7.64; *t*(21) = 6.21, *p* < 0.001). Session 2 data were collected from 23 placebo non-responders who were re-randomized to bupropion and subsequently classified as responders (*n* = 8) or non-responders (*n* = 15). Baseline HAMD scores did not differ (responders: 30.25 ± 6.50; non-responders: 31.53 ± 7.38), but HAMD scores were lower in responders (9.88 ± 3.18) vs. non-responders (25.54 ± 7.42) at the final sesssion *t*(19) = 5.62, *p* < 0.001.

### Signal Detection Analyses

Signal detection analyses were initially conducted for all participants with usable data, regardless of randomization to placebo vs. bupropion or responder status. Detailed results are in Table S2. In summary, a response bias was observed in both sessions. In Session 1, regressing response bias on *Block* revealed larger values in block 3 vs. 1 (coefficient = 0.08, SE = 0.04, *t*-value = 2.15, *p* = 0.033), providing evidence of learning. In Session 2, a similar regression revealed no differences between blocks (*p*s > 0.15), but one-sample *t*-tests confirmed that response bias was above zero in every block (*t*s > 4.65, *p*s < 0.001, Cohen’s d values > 0.69). Linear regressions of discriminability on *Block* did not yield any significant effects in Session 1 or 2 (*p*s > 0.09), but all values were well above zero. Internal consistency, assessed by comparing odd vs. even trials, was high for response bias (Session 1: SB = 0.85; Session 2: SB = 0.84) and discriminability (Session 1: SB = 0.88; Session 2: SB = 0.91). Retest reliability was modest but significant for response bias (*r* = 0.59) and discriminability (*r* = 0.49), *ps* < 0.004.

### Relationships Among Behavioral Variables

**Figure 2:**
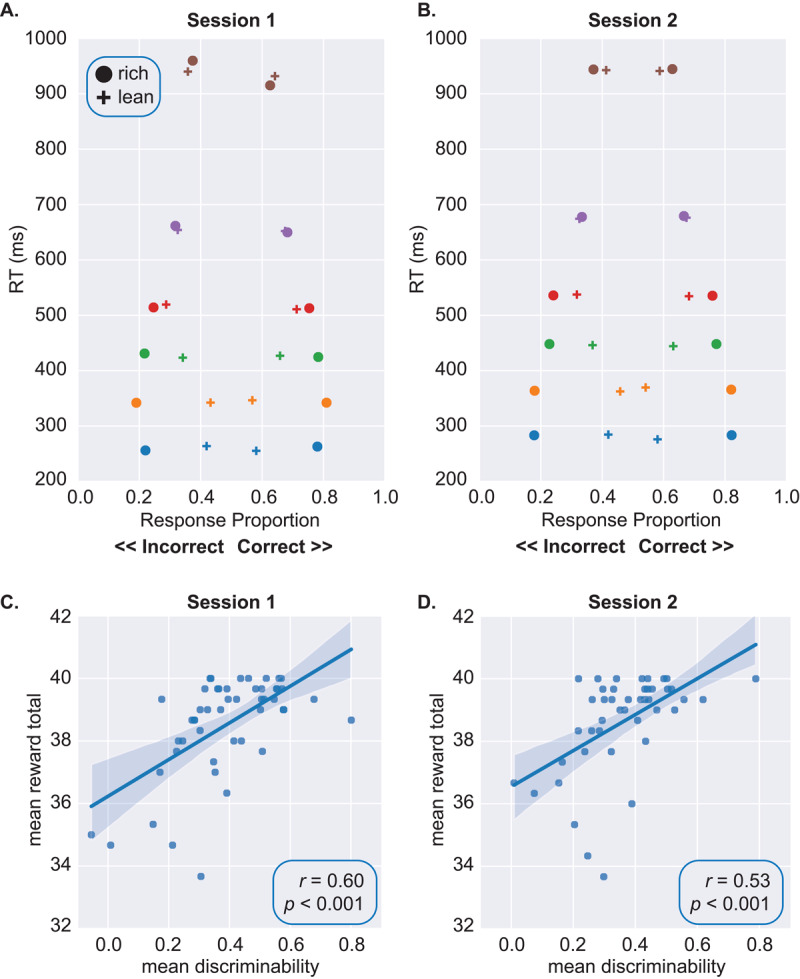
Quantile Probability Plots and Relationship Between Reward Totals and Discriminability. *Note*. Top row shows quantile probability plots in (A) Session 1 and (B) Session 2. RT quantiles are color coded: .10 (blue), .30 (orange), .50 (green), .70 (red), .90 (purple), and .995 (brown). Data for rich and lean stimuli are shown in circles and crosses, respectively. Bottom row shows the relationship between mean reward totals and discriminability at the block level in (C) Session 1 and (D) Session 2.

Figures 2 A and 2B show quantile probability plots. The RT distributions are skewed: the gaps between the 99.5^th^ and 90^th^ quantiles, and between the 90^th^ and 70^th^ quantiles, are larger than the gaps between lower quantiles. The plots also show that the rich > lean accuracy effect is larger for faster RTs: the horizontal distance between the rightmost circles and crosses, which captures the difference in proportion correct responses to the rich vs. lean stimuli, is larger for shorter RTs (e.g., .10 quantile in blue) vs. longer RTs (e.g., 0.995 quantile in brown).

For each participant we coded RTs < = 0.3 quantile as fast and all other RTs as slow (Dillon et al., 2022; Lawlor et al., 2020), and then ran logistic regressions on accuracy with factors *Stimulus* (rich, lean), *ResponseSpeed* (fast, slow), and their interaction; participants were treated as random effects (accuracy ~ stimulus * response_speed + (1|subject)). These models returned strong *Stimulus* × *ResponseSpeed* interactions (Session 1: coefficient = –1.08, SE = 0.08, *Z* = –12.95; Session 2: coefficient = –1.26, SE = 0.09, *Z* = –14.27). Follow-up analysis showed that while the rich > lean accuracy effect is always present, it is stronger for fast RTs (*Z*s > 18) vs. slow RTs (*Z*s < 7). Second, lean accuracy is lower for fast vs. slow RTs (*Z*s < –10) but rich accuracy is higher for fast vs. slow RTs (*Z*s *>* 7). Thus, participants disproportionately respond “rich” when RTs are short, but are more even-handed when RTs are long.

Strong positive relationships between reward totals and discriminability emerged in Sessions 1 (Figure 2C) and 2 (Figure 2D). By contrast, reward totals were not correlated with response bias in Session 1 (*r* = –0.02) or 2 (*r* = 0.01). Meng tests (Meng et al., 1992) confirmed that reward totals were more strongly related to discriminability than response bias in both sessions (*Z*s > 2.4, *p*s < 0.015).

### HDDM

#### Posterior Distributions

**Figure 3:**
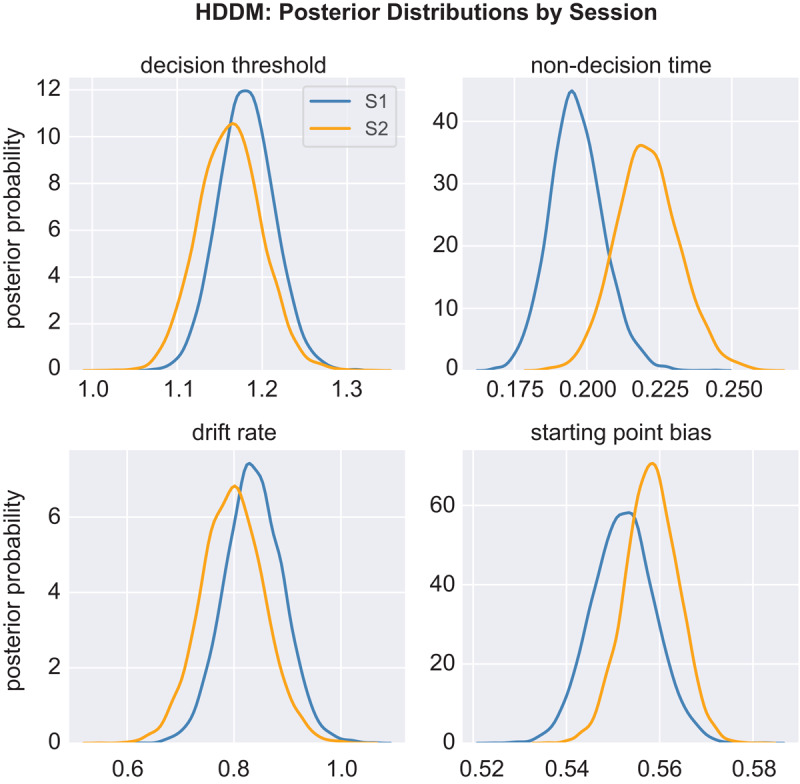
HDDM: Posterior Distributions by Session. *Note*. Posterior distributions from Sessions 1 (blue) and 2 (orange).

Figure 3 shows HDDM posterior distributions. Three findings are noteworthy. First, the starting point is biased away from the midline (0.5) and towards the rich boundary (coded 1), consistent with a high number of “rich” responses when RT is fast. Second, drift rate is slow. We have applied the HDDM to six prior PRT datasets—two MDD samples and two healthy control samples in Lawlor et al., 2020; one socially anxious and one healthy control sample in Dillon et al., 2022)—and the mean drift rate has always exceeded 1, whereas here it is well below 1. Indeed, while the posterior distributions for threshold, starting point, and non-decision time in Figure 3 are all similar to those observed in the large (*n* = 258) MDD sample tested in Lawlor et al. (2020), the drift rates are markedly lower here. Third, changes across sessions are modest. We computed the percent overlap for Session 1 and 2 posterior distributions, referred to as a *q*-value, and found less than 5% overlap only for non-decision time (*q* = 0.044).

#### Posterior Predictive Checks

HDDM parameters were used to simulate 500 datasets, and the observed data—including the percentage of “rich” responses and the RT means, SDs, and 0.1/0.3/0.5/0.5/0.9 quantiles—all fell within 95% credible intervals based on the simulations, indicating that the HDDM fit well.

**Figure 4:**
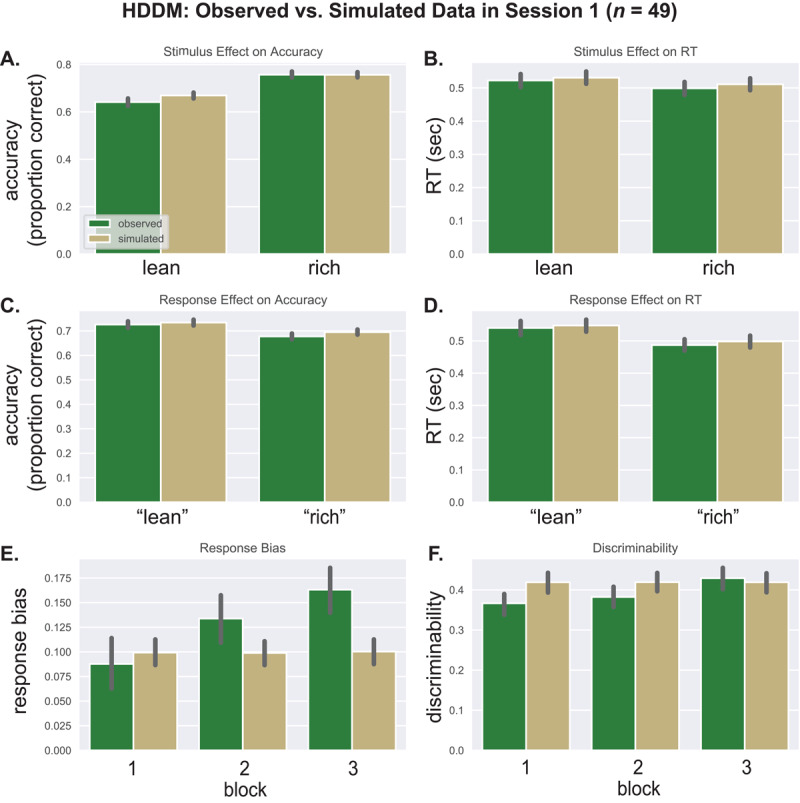
HDDM: Observed vs. Simulated Data in Session 1. *Note*. Session 1: observed data vs. HDDM simulations. Results for (A) the stimulus effect (rich/lean) on accuracy, (B) the stimulus effect on RT, (C) the response effect (“rich”/ “lean”) on accuracy, (D) the response effect on RT, (E) response bias, and (F) discriminability.

To confirm this, we plotted simulated against observed data. Figure 4 shows a close correspondence between observed and simulated accuracy and RT in Session 1, whether the data were binned by stimulus (Figure 4a, 4b), or response (Figure 4c, 4d). Next we binned the simulated data in 100 trial blocks to compute response bias and discriminability. Figure 4e shows that simulated response bias underpredicted observed bias in blocks 2 and 3; moreover, the simulated response bias did not vary by block, as expected because HDDM parameters were estimated across blocks. The correspondence between simulated and observed discriminability was good (Figure 4f). Session 2 simulations yielded similar results (Supplemental Figure S2).

#### Relationship with Standard PRT Metrics

Results of regressing mean (across blocks) response bias and discriminability on HDDM parameters are in Table S3. Starting point bias was the only reliable predictor of response bias in Sessions 1 and 2 (*p*s < 0.001). Discriminability was strongly predicted by drift rate and decision threshold in both sessions (*p*s < 0.001).

#### Psychometrics

Table 1 shows internal consistency. For the HDDM, Spearman-Brown coefficients exceeded 0.80, except for Session 2 starting point bias computed on the first vs. second half of trials (SB = 0.651). Average Spearman-Brown coefficents were higher for parameters estimated on odd vs. even trials (Session 1: 0.929; Session 2: 0.927) than first vs. second half of trials (Session 1: 0.865; Session 2: 0.812). Retest reliability (Pearson *r*-values) of HDDM parameters is in Table S4 and ranged from 0.36 (non-decision time) to 0.67 (threshold), all *p*s < 0.05.

**Table 1:** Internal Consistency of the HDDM and the Belief Model.


PARAMETER	ODD/EVEN TRIALS	FIRST HALF/SECOND HALF

**HDDM: Session 1**		

Threshold (*a*)	0.982	0.890

Non-decision time (*t*)	0.899	0.866

Drift rate (*v*)	0.914	0.894

Starting bias (*z*)	0.919	0.807

**HDDM: Session 2**		

Threshold (*a*)	0.982	0.893

Non-decision time (*t*)	0.966	0.833

Drift rate (*v*)	0.920	0.873

Starting bias (*z*)	0.841	0.651

**Belief Model: Session 1**		

Reward sensitivity	–0.079	0.457

Instruction sensitivity	0.883	0.800

Learning rate	–0.118	0.325

Belief	–0.142	0.555

Initial bias	–0.090	0.594

**Belief Model: Session 2**		

Reward sensitivity	0.010	0.557

Instruction sensitivity	0.856	0.757

Learning rate	0.010	0.396

Belief	0.380	0.717

Initial bias	0.084	0.609


*Note*. Values are Spearman-Brown coefficients, computed by comparing parameter estimates computed for odd vs. even trials (“Odd/even trials”), or for the first vs. second half of the task (“First half/second half”), separately by session for the HDDM and Belief models.

#### RLDDM

The data were better fit by the HDDM than by the RLDDM. This conclusion is supported by lower DIC values in Session 1 (HDDM: 8080.07; RLDDM: 8776.43) and Session 2 (HDDM: 7224.18; RLDDM: 7799.77), and by posterior predictive checks. We used RLDDM parameters to generate 50 simulated datasets and again plotted simulated data alongside observed data. Supplemental Figures S3 and S4 show poorer correspondence for the RLDDM vs. HDDM. In particular, the RLDDM underpredicted accuracy and discriminability. The RLDDM did capture the across-block increase in response bias. However, block 1 simulated response bias was negative in both sessions, which was not seen in the actual data, and a quantitative fit between simulated and actual values was not evident until block 3.

### Belief Model

#### Parameter Values

**Figure 5:**
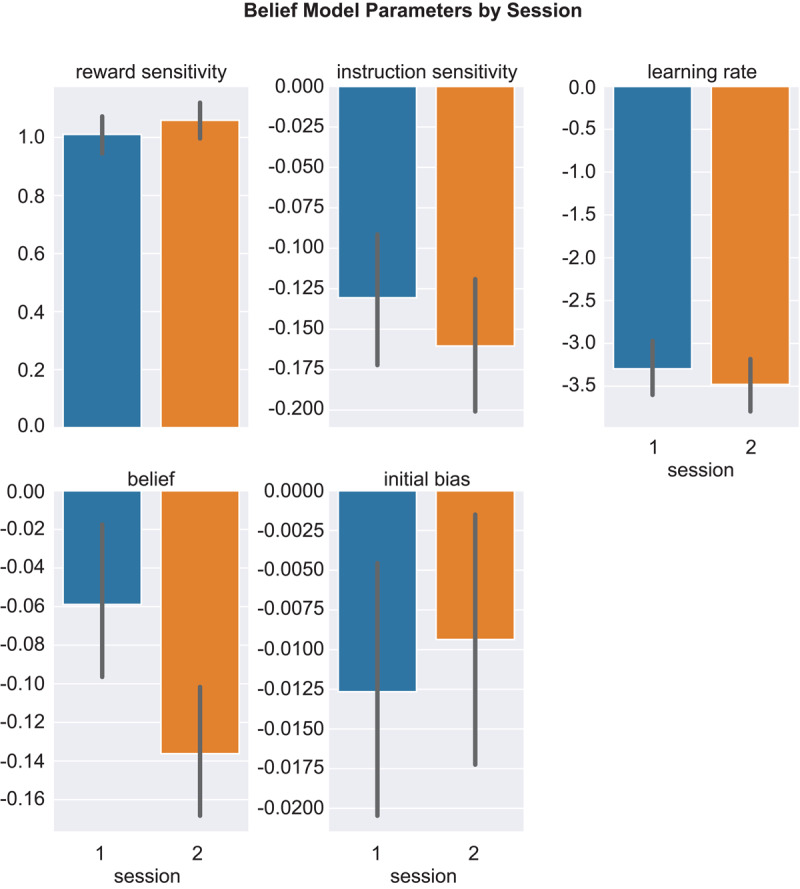
Belief Model Parameters by Session. *Note*. Belief model parameters in Sessions 1 (blue) and 2 (orange). Error bars show S.E.M.

Figure 5 shows belief model parameters. Changes across the sessions were modest, with paired *t*-tests returning no significant differences (*p*s > 0.41).

#### Relationship with Standard PRT Metrics

Results of regressing mean (across blocks) response bias and discriminability on belief model parameters are in Table S5. Session 1 response bias was significantly predicted by all model parameters but initial bias; Session 2 response bias was predicted by reward sensitivity and learning rate (*ps* < 0.001). Discriminability was predicted by instruction sensitivity and belief in both sessions (*p*s < 0.001), and was negatively predicted by learning rate in Session 2 (*p* = 0.040).

#### Posterior Predictive Checks

**Figure 6:**
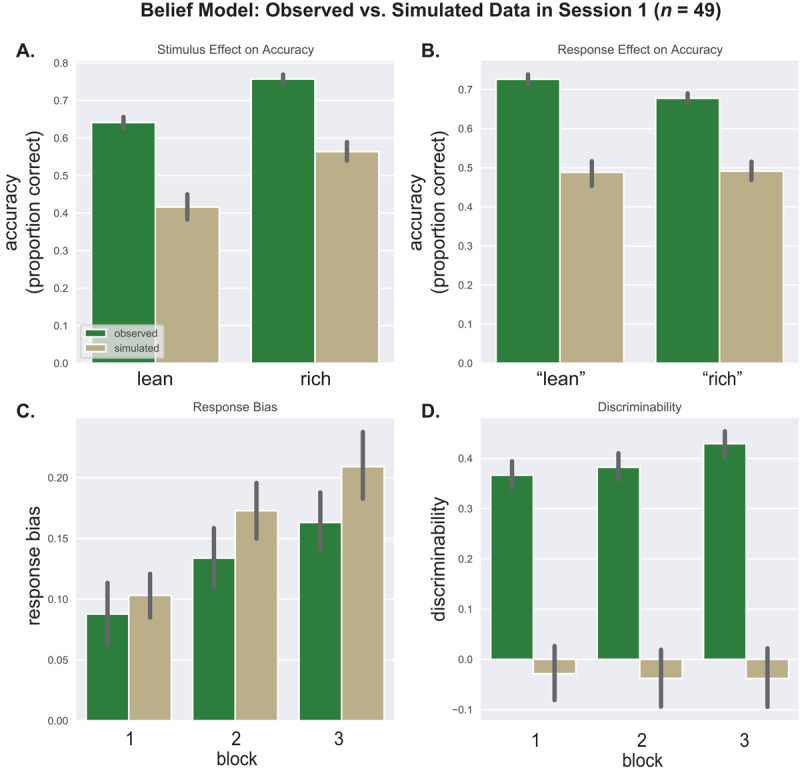
Belief Model: Observed vs. Simulated Data in Session 1 (n = 49). *Note*. Session 1: observed data vs. belief model simulations. Results are shown for (A) the stimulus effect (rich/lean) on accuracy, (B) the response effect (“rich”/ “lean”) on accuracy, (C) response bias, and (D) discriminability.

Figure 6 shows actual vs. simulated Session 1 data for the belief model; because this model does not account for RT, only choice results are shown. The model captures the increase in response bias over the blocks, and higher accuracy for the rich vs. lean stimulus. However, it does not reproduce higher accuracy for “lean” vs. “rich” responses, accuracy is generally too low in the simulated data, and the model sharply underpredicts discriminability. Results for Session 2 are similar (Figure S5).

#### Psychometrics

Table 1 shows that Spearman-Brown coefficients for belief model parameters ranged from –0.142 to 0.883; the average coefficent was higher for parameters estimated using the first vs. second half of trials (Session 1: 0.546; Session 2: 0.607) than for odd vs. even trials (Session 1: 0.091; Session 2: 0.268). The mean ± SD Spearman-Brown value, averaged across parameters, sessions, and both methods for splitting up the data, was lower for the belief model (0.378 ± 0.346) vs. the HDDM (0.883 ± 0.077). Retest reliability (Pearson *r*-values) for belief model parameters is in Table S4; values ranged from –0.46 (initial bias) to 0.33 (instruction sensitivity), with only the initial bias value being significant.

### Prediction of Responses to Placebo and Bupropion

ANOVAs comparing response bias and discriminability in placebo responders vs. non-responders yielded no significant effects in either session (*p*s > 0.09). Similar results emerged for the HDDM: across Sessions 1 and 2, the posterior distributions for placebo responders vs. non-responders always showed extensive overlap (*q*-values > 0.18). The belief model returned no significant effects in Session 2. However, in Session 1 eventual placebo responders showed lower reward sensitivity (responders: 0.57 ± 0.44; non-responders: 1.14 ± 0.40; *p* < 0.001, *d* = 1.40) and higher learning rates (responders: –1.04 ± 1.67; non-responders: –4.05 ± 1.68; *p* < 0.001, *d* = 1.80) than non-responders.

**Figure 7:**
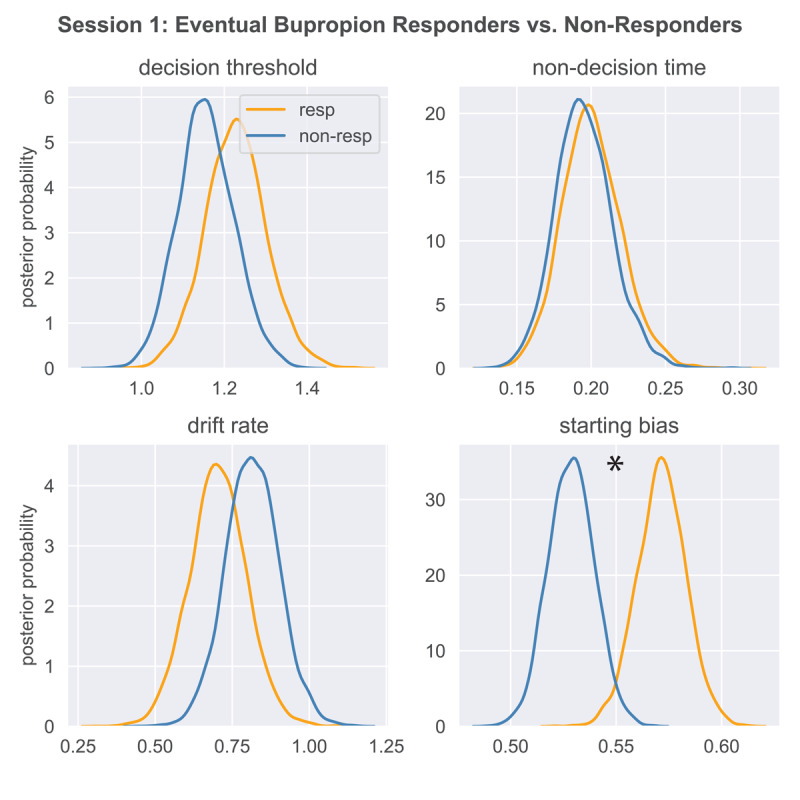
Session 1 HDDM Parameters in Eventual Bupropion Responders vs. Non-responders. *Note*. Asterisk marks < 1% overlap of posterior distributions between groups.

ANOVAs on response bias and discriminability in placbo non-responders re-randomized to bupropion yielded no differences between bupropion responders vs. non-responders in Session 1 or 2 (*p*s > 0.12). Similarly, between-group comparisons of belief model parameters were non-significant in both sessions (*p*s > 0.15). By contrast, as shown in Figure 7, the HDDM revealed a difference in Session 1 starting point bias, which was higher in the (eventual) bupropion responders vs. non-responders (q-value < 0.01). In Session 2, this difference was weaker (q-value = 0.10) but a group difference in decision threshold (responders > non-responders) emerged (q-value = 0.04). Figure S6 shows mean starting point bias values for bupropion responders vs. non-responders in Session 1. Six non-reponders have values below those of every responder, suggesting that starting point bias in the PRT might be useful for predicting bupropion (non-) response in individuals.

Conceptually, the analyses in this section may be more easily understood in a logistic regression framework, where the initial placebo response (0 = non-responder, 1 = responder) might be predicted by Session 1 response bias, discriminability, belief model parameters, or HDDM parameters. We ran these analyses and results very closely resembled the ANOVA outcomes: placebo response was not predicted by Session 1 response bias, discriminability, or HDDM parameters, but it was negatively predicted by reward sensitivity (coefficient = –4.16, SE = 1.47, *Z* = –2.82, *p* = 0.004) and positively predicted by learning rate (coefficient = 0.87, SE = 0.27, *Z* = 3.29, *p* = 0.001) from the belief model. Logistic regressions were also run to predict responses to bupropion (in placebo non-responders) with the same Session 1 variables. The only reliable predictor was Session 1 starting point bias, from the HDDM (coefficient = 29.73, SE = 13.30, *Z* = 2.24, *p* = 0.025). Finally, logistic regressions predicting responses to bupropion (in placebo non-responders) with Session 2 variables were also run. These returned no significant effects. Thus, across the ANOVA and logistic regression analyses, placebo responses were negatively predicted by reward responsivity but positively predicted by learning rate (both from the belief model) in Session 1, whereas responses to bupropion (following initial non-response to placebo) were predicted by Session 1 starting point bias from the HDDM.

## Discussion

This PRT study yielded several findings. First, we again found that response bias varies with RT: the rich > lean accuracy difference was larger for faster vs. slower RTs. When participants respond quickly, they often respond “rich” regardless of which stimulus is shown; when they respond more slowly, the stimulus effect on accuracy (and thus response bias) is reduced. Second, reward totals were better predicted by discriminability than by response bias. Third, the data were well-fit by the HDDM. There was close correspondence between the data and HDDM simulations, although the HDDM did not capture increased response bias over blocks. Internal consistency of HDDM parameters was good to excellent, and retest reliability was adequate. Finally, comparing the HDDM parameters to those from prior reports (Dillon et al., 2022; Lawlor et al., 2020) revealed that drift rate was low in this depressed sample, consistent with the proposal that slow evidence accumulation is a broad marker of psychopathology (Sripada & Weigard, 2021; but see Dillon et al., 2022). Fourth, the belief model captured the increase in response bias over blocks, but this model produced lower accuracy and discriminability than was observed, and its parameters were psychometrically weaker: the internal consistency and retest reliability of belief model parameters was poorer than for HDDM parameters. Fifth, the models provided insight into prediction of responses to placebo and bupropion. These results must be interpreted cautiously due to very small samples. Nonetheless, at baseline the belief model indicated that eventual placebo responders showed weaker reward responsivity but higher learning rates than non-responders. The HDDM revealed stronger starting point biases in placebo non-responders who eventually responded to bupropion. Overall, these findings provide insight into cognitive processes that are recruited by the PRT and affected by depression, while also providing leads for larger studies focused on predicting placebo responses.

### Cognition in the PRT

Many studies use response bias in the PRT as a behavioral test of reward system function and a probe for anhedonia, but few have examined the nature of response bias or closely examined discriminability. This paper is the third to do so (Dillon et al., 2022; Lawlor et al., 2020), and all three yielded similar results that make two points about PRT performance.

First, quantile-probability plots consistently show that the response bias effect is strong when RTs are fast but is weaker, if not absent, for slower responses. As explicated by White and Poldrack (2014), this dependency between accuracy and RT marks a true response bias: on many trials, participants quickly press “rich” regardless of which stimulus is shown. In other words, participants exhibit a response-outcome association (Rescorla, 1992)—because “rich” responses are likely to be rewarded, on many trials they make fast “rich” responses. Second, the quantile-probability plots and correlational analyses show that there is more to the PRT than response bias. Although the rich > lean accuracy effect is strong for the fastest 30% of responses, on the remaining trials the response bias effect is small and accuracy for the rich and lean stimuli is similar. This surprising but consistent result indicates that an account of PRT performance should not stop at response bias, but should also explain the many deliberate, more even-handed responses observed. The importance of this issue is underlined by the fact that reward totals were again better predicted by discriminability than by response bias: if a participant has good discriminability, it is to their advantage to make unbiased, accurate responses.

The HDDM can account for discriminability and response bias. Variation in discriminability is explained by drift rate and threshold, with other parameters making much smaller contributions. This is sensible because accuracy in the DDM is governed by the speed with which participants assemble the evidence needed to respond and the distance between the thresholds that the accumulating evidence must cross; fast evidence accumulation (high drift rate) plus widely spaced boundaries (high decision thresholds) yields accurate responding. The HDDM explains response bias with starting point bias: the accumulation process begins closer to the “rich” boundary, such that little evidence for the rich stimulus needs to accumulate for that response to be made. Consequently, there are more fast “rich” vs. “lean” responses.

With three parameters—drift rate, decision threshold, and starting point bias—the HDDM can thus account for PRT data. However, the HDDM estimates the average starting point bias and does not capture the increase in response bias over blocks. This reflects the fact that that the DDM does not model learning: it can account for bias, but not for how a bias forms. Also, while it is is encouraging to consistently find a close relationship between response bias in the PRT and starting point bias in the HDDM, the one-to-one nature of this relationship limits its explanatory value: how the starting point bias develops and what it reflects with regard to underlying neural mechanisms is unclear. Focusing on pre-stimulus value signals in motor regions (Cisek, 2007) might be a useful first step towards identifying neural mechanisms that underlie starting point bias.

To summarize, the PRT elicits two patterns of behavior. First, the asymmetric reinforcement induces a response bias: participants learn that one response is rewarded more often than the other, and so on many trials they simply press the “rich” key very quickly. The stimuli contribute little to this behavior. The second pattern, however, is that on many trials participants respond more slowly and accuracy for the rich and lean stimuli is similar. This aspect of behavior is reflected in discriminability, it is captured by drift rate (and threshold) in the HDDM, and it has received much less attention. However, this manuscript and our prior papers (Dillon et al., 2022; Lawlor et al., 2020) have shown clearer effects of psychopathology on drift rate and discriminability than on the starting point bias and response bias. Also, all three studies show that drift rate and discriminability predict what participants care most about—namely, cumulative reward totals. In short, the emphasis on discriminating between two similar stimuli—which is central to the PRT but uncharacteristic of most RL tasks—appears valuable for understanding how psychopathology affects cognition.

### Depressive Cognition

The current sample generated the lowest drift rate we have seen in our PRT studies, which is striking as the values for all other HDDM parameters were similar to those in a large (*n* = 258) MDD sample (Lawlor et al., 2020). Consequently, despite the lack of a control group, these data again reveal slow evidence accumulation in MDD, similar to what is seen in other forms of psychopathology (Sripada & Weigard, 2021). The underlying cause of slow evidence accumulation in MDD is unknown—possibilities include low cortical dopamine (Beste et al., 2018) or disrupted white matter tracts (Wang et al., 2014)—and this is a target for future work.

Surprisingly, there was no evidence of a blunted response bias in this treatment-seeking sample: in both sessions, a response bias emerged and the HDDM’s starting point was shifted towards the “rich” boundary. Interpretation must be somewhat tentative given the lack of controls, but this MDD group was sensitive to the reinforcement. This raises the point that while depression can negatively affect reward function, the extent of the disruption varies widely; indeed, even when controls show a stronger response bias than depressed adults, the depressed group often still shows the effect (Pizzagalli et al., 2008c). Given increased appreciation of the fact that depression is heterogeneous, that mental health is often better conceptualized as continuous rather than dichotomous, and that many effect sizes in psychology research are smaller than originally supposed, going forward it may be productive to collect larger PRT datasets, identify individuals with anomalous results (e.g., no response bias), and then study those datasets to learn more about underlying mechanisms and their relationship with MDD.

### Model Comparison

The HDDM models the PRT as a perceptual decision-making task in which one response is more frequent than the other, whereas the belief model envisions the PRT as an RL task in which participants use PEs to update values assigned to (stimulus, action) pairs. These distinct conceptualizations might cause analysts to prefer one model over the other. The current analysis aimed to inform such choices through simulations and by examining psychometrics.

Overall, the results were more favorable to the HDDM. HDDM simulations were similar to the data with respect to accuracy, RT, and discriminability, and the HDDM simulated a response bias, although it did not vary by block. HDDM parameters showed good to excellent internal consistency and adequate retest reliability, and the stability of relationships between response bias, discriminability, and HDDM parameters was encouraging. Response bias was selectively related to starting point bias in the HDDM, whereas discriminability was strongly related to drift rate and, to a lesser extent, threshold (Lawlor et al., 2020; Dillon et al., 2022). These results indicate that the HDDM captures key features of PRT data, with the parameters being sufficiently stable within and across sessions to study individual differences.

The picture was more mixed for the belief model. This model captured the response bias increase over blocks and higher accuracy for the rich vs. lean stimulus. However, it did not capture the response effect on accuracy, simulated accuracy was too low, and simulated discriminability was much lower than actual discriminability. Also, relationships between belief model parameters and signal detection metrics were less selective and stable than for the HDDM. For example, in Session 1 response bias was predicted by every belief model parameter except initial bias, whereas in Session 2 it was predicted only by reward sensitivity and learning rate. Along with the weaker psychometric results, this variability suggests cautious interpretation of belief model parameters.

Because the belief model involves learning from trial-by-trial feedback, which the HDDM does not, its psychometric properties may be inherently less stable. We attempted to address this concern by assessing internal consistency with first-half vs. second-half comparisons, so as not to disrupt trial-level sequences. This was helpful in that internal consistency for belief model parameters was higher using the “by-halves” approach vs. the odd/even approach, but internal consistency was still worse for belief model vs. HDDM parameters with the by-halves approach. Also, the by-halves approach introduces temporal delays that may negatively affect internal consistency—indeed, the consistency of HDDM parameters was lower using the by-halves method. Recall that all participants received placebo or bupropion between sessions, which could affect the assessment of test-retest reliability. This raises the possibility that poorer reliability for the belief model is due to greater sensitivity to the interventions. This seems unlikely given that paired *t*-tests examining session effects on belief model parameters were all non-significant. Nevertheless, fairly assessing the consistency of RL models is non-trivial and should be a focus going forward, especially because many clinically-oriented hypotheses assume lasting disruptions to reward sensitivity and RL; psychometrically stable RL measures are needed to test these hypotheses (Brown et al., 2021).

### Treatment Prediction

These data are from a project examining prediction of placebo responses; the core hypothesis was that individuals with stronger reward system function were more likely to be placebo responders. Unfortunately, the number of placebo responders with usable PRT data was small, limiting the conclusions that can be drawn from prediction analyses. Indeed, effect size estimates for responder vs. non-responder differences in belief model parameters are implausibly large (e.g., *d* = 1.40); with small samples, only large effects will be significant and such effects are nearly always smaller when studied in larger samples (Button et al., 2013). The same concern applies for the HDDM parameters.

Nevertheless, because the results might inform better-powered work, we describe them here. First, the belief model indicated that baseline reward responsivity was lower and learning rate was higher in (eventual) placebo responders vs. non-responders. Second, the HDDM indicated that baseline starting point bias was higher in (eventual) bupropion responders vs. non-responders; this analysis included only placebo non-responders, to identify predictors of bupropion response unconfounded with placebo.

With respect to the belief model, lower reward responsivity in placebo responders contradicts the core hypothesis but higher learning rates are broadly consistent with enhanced reward system function. Higher learning rates indicate a stronger effect of recently received rewards on value updating. Thus, these findings may fit with theories emphasizing reward-based learning processes in placebo effects (Lee et al., 2015; Schmidt et al., 2014; Turi et al., 2017).

The HDDM revealed larger starting point biases in bupropion responders vs. non-responders. There is some precedent, as prior work found that reward system function positively predicted responses to pramipexole, a dopamine agonist (Whitton et al., 2020). Furthemore, adults with MDD who responded to bupropion (after failing to respond to sertraline) showed a higher pre-treatment response bias in the PRT than bupropion non-responders (Ang et al., 2020). These results indicate that larger PRT studies of placebo prediction may be warranted.

## Conclusion

The PRT is a widely used probe of reward system function, with weak response bias often taken as a marker of anhedonia. Here we provided a detailed analysis that clarifies the nature of response bias and highlights the importance of discriminability. We also applied two computational models to the data. The HDDM accounted for response bias and discriminability and showed strong psychometric properties. The belief model performed less well psychometrically, but—unlike the HDDM—it reproduced the increase in response bias over blocks. Finally, the study provided preliminary evidence that the PRT may be useful for predicting treatment responses, with perhaps the strongest result being a larger baseline starting point bias in those who subsequently responded to bupropion. This result emerged from very small samples and awaits replication, but it supports the idea that bupropion may be most effective in depressed adults whose reward system function is relatively intact. Overall, these data provide insight into the PRT, into depressive cognition, and into mechanisms of behavior that can be captured by computational models.

## Additional File

The additional file for this article can be found as follows:

10.5334/cpsy.108.s1 Supplemental Material. Figures S1–S6 and Tables S1–S5.
