# Supplementary material for: Using Drift Diffusion and RL Models to Disentangle Effects of Depression On Decision-Making vs. Learning in the Probabilistic Reward Task
Source: Comput Psychiatr. 2024 May 3;8(1):46–69. doi: 10.5334/cpsy.108 (PMC11104335; doi:10.5334/cpsy.108)
Supplement: Supplemental Material. — Figures S1–S6 and Tables S1–S5. [file cpsy-8-1-108-s1.pdf]

**Supplemental Material for**

*Computational analyses of reward-based decision-making in depressed adults*

## Exclusion Criteria

The exclusion criteria were as follows: (a) pregnancy or childbearing potential without a medically accepted contraceptive; (b) serious suicide or homicide risk; (c) unstable medical illness; (d) any of the following DSM-IV diagnoses—organic mental disorders, substance use disorders within the last year, psychotic disorders, bipolar disorder, acute bereavement, severe borderline or antisocial disorder, eating disorder, current primary diagnosis of panic disorder, social anxiety disorder, posttraumatic stress disorder, generalized anxiety disorder, or obsessive compulsive disorder, mood congruent or incongruent psychotic features; (e) history of abuse of stimulants or opiates; (f) current use of antipsychotics, anticonvulsants, stimulants, antidepressants, or augmenting agents [e.g., St. John's Wort]; (g) use of any investigational psychotropic drug in the last year; (h) non-response to two or more antidepressant trials of adequate dose and duration, per the ATHQ, over the last five years; (i) history of inadequate response to or poor tolerability of bupropion; (j) concomitant psychotherapy for depression; (k) current or prior treatment with vagal nerve stimulation, electroconvulsive therapy, or transcranial magnetic stimulation; or (l) red/green colorblindness (due to a task used in the PET/MRI scan).

**Figure S1**

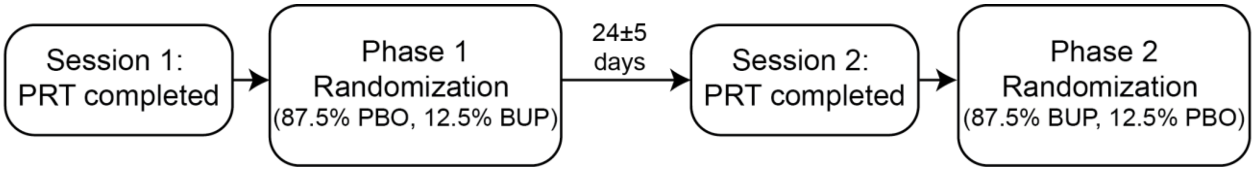


*Note*. Simplified protocol summary. PRT data were acquired immediately prior to Phase 1 randomization to placebo (PBO) or bupropion, and again immediately before Phase 2 re-randomization. All participants were diagnosed with Major Depressive Disorder, and Phase 1 randomization was disproportionately to the placebo condition given the goal to identify predictors of placebo response.

**Figure S2**

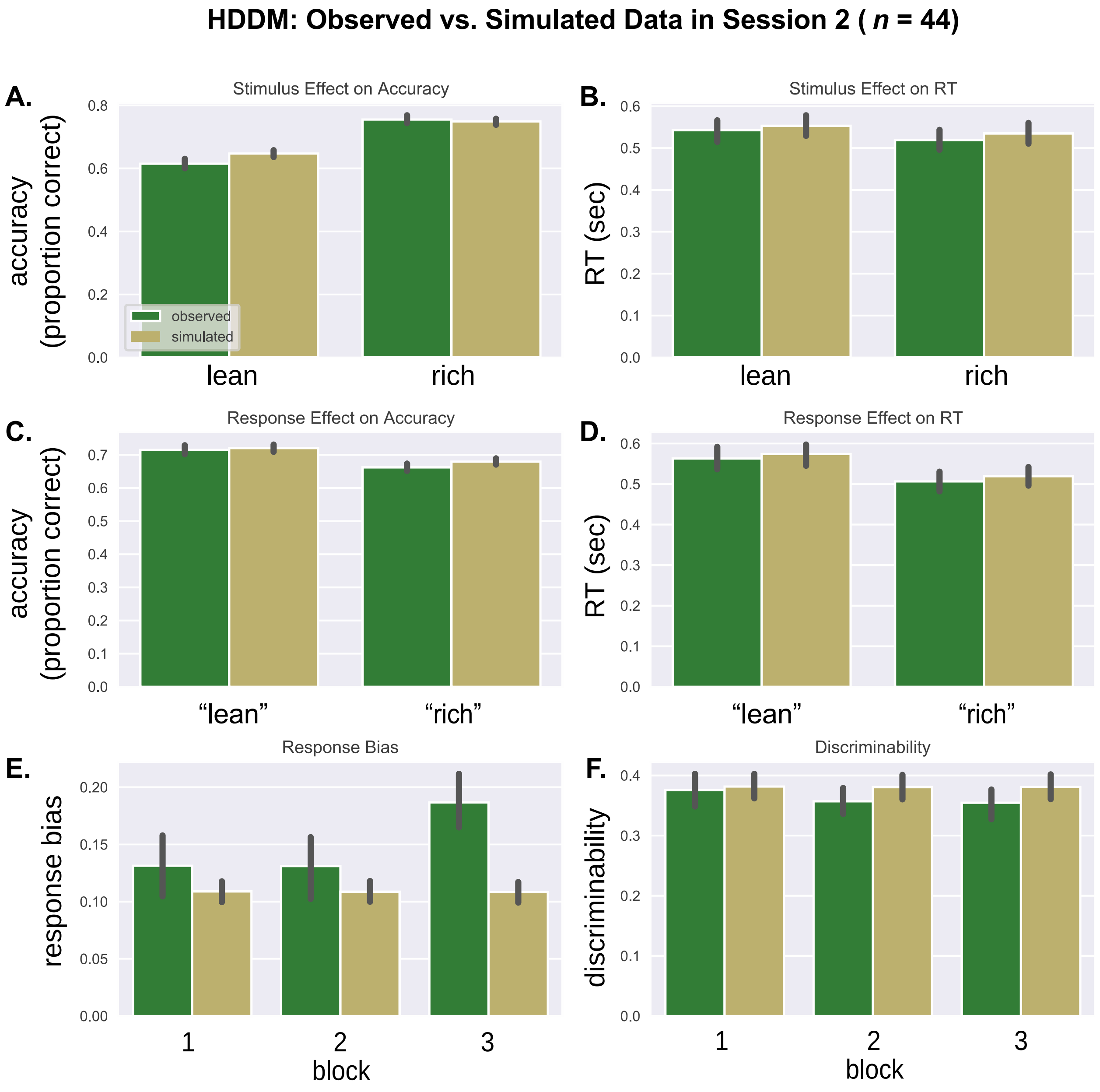


*Note*. Observed data from Session 2 vs. data from 500 simulations, generated using the HDDM. Results are shown for (A) the stimulus effect (rich/lean) on accuracy, (B) the stimulus effect on RT, (C) the response effect ("rich"/ "lean") on accuracy, (D) the response effect on RT, (E) response bias, and (F) discriminability.

**Figure S3**

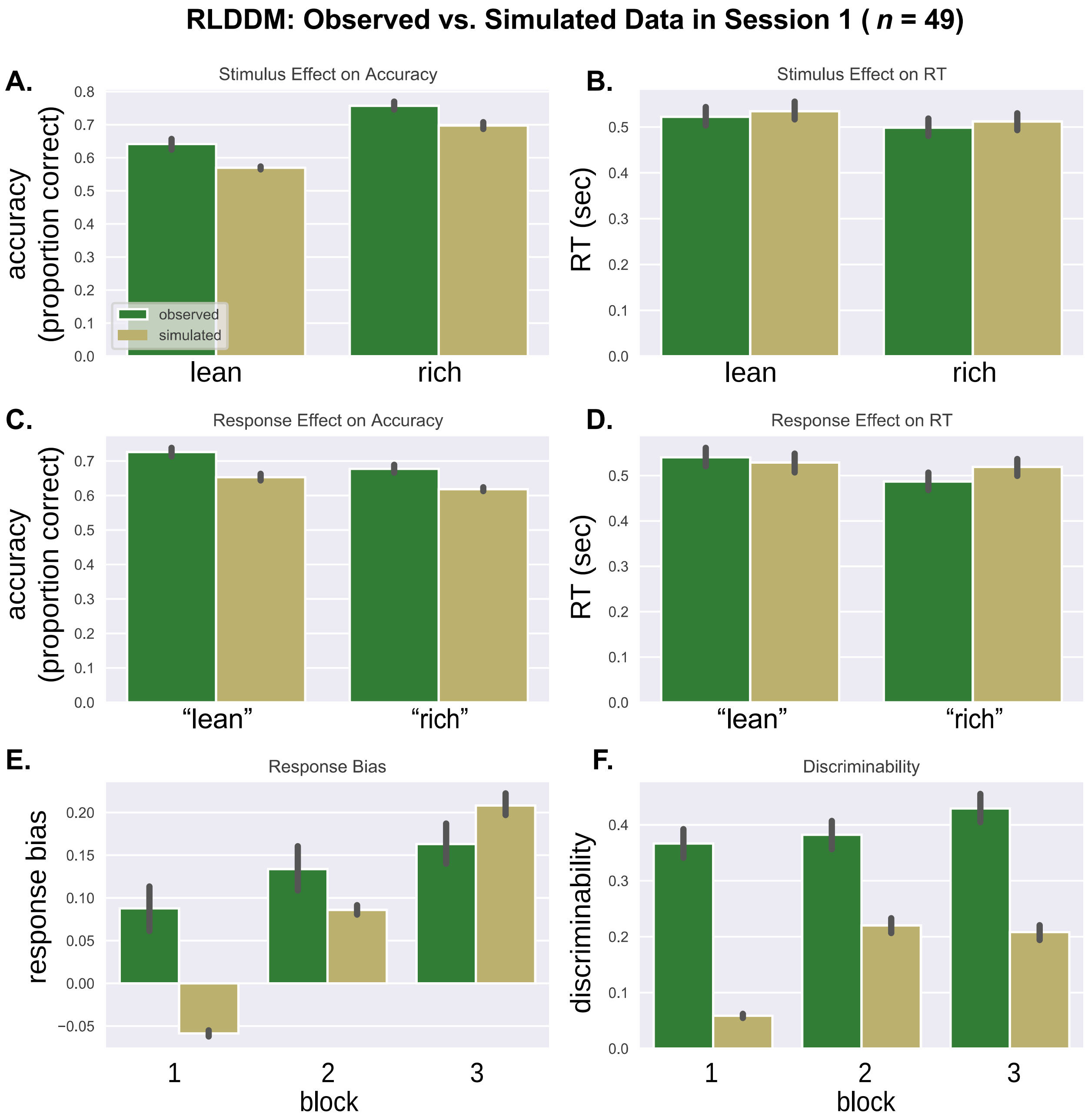


*Note*. Observed data from Session 1 vs. data from 50 simulations, generated using the RLDDM. Results are shown for (A) the stimulus effect (rich/lean) on accuracy, (B) the stimulus effect on RT, (C) the response effect (“rich”/ “lean”) on accuracy, (D) the response effect on RT, (E) response bias, and (F) discriminability.

**Figure S4**

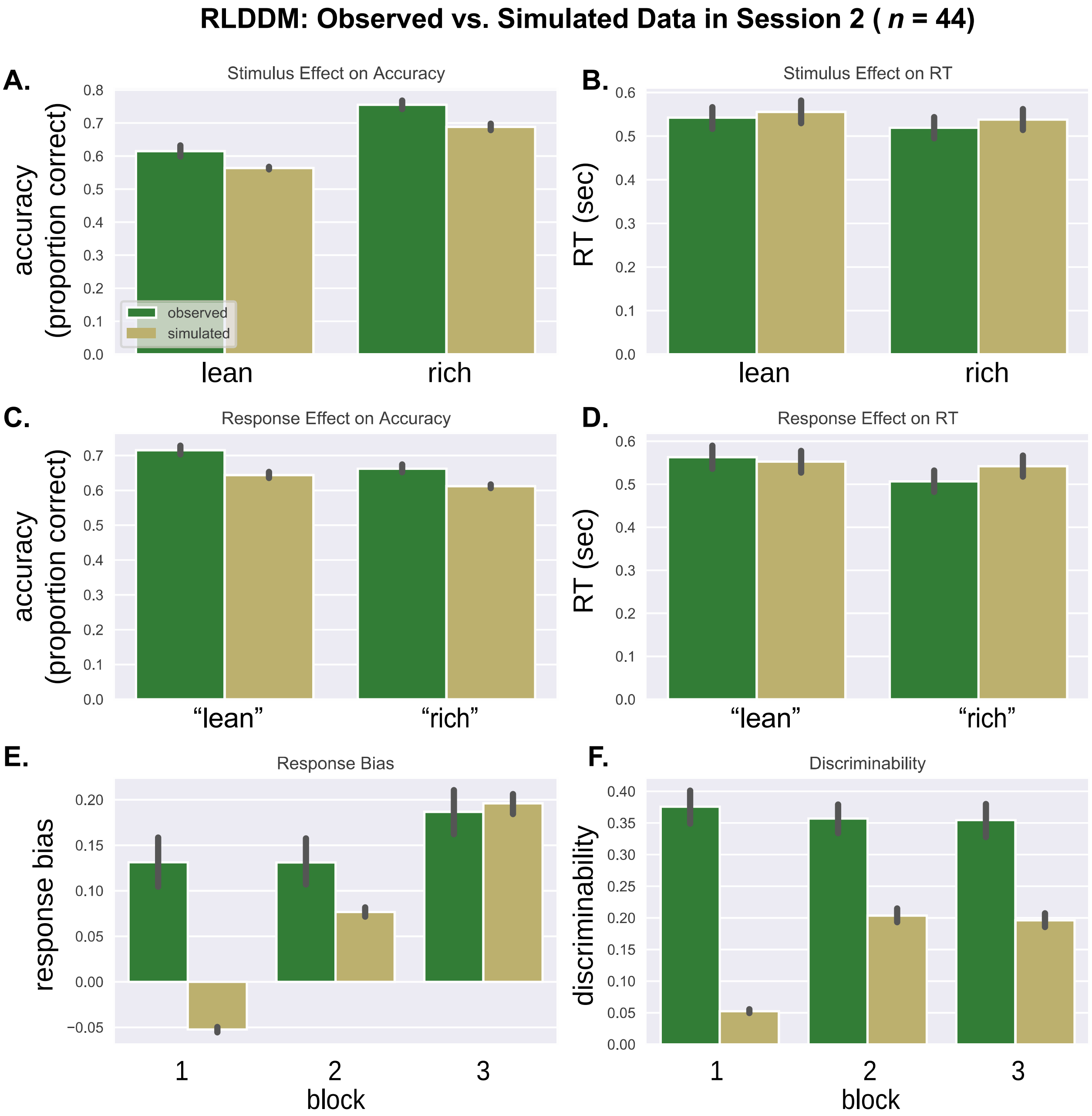


*Note*. Observed data from Session 2 vs. data from 50 simulations, generated using the RLDDM. Results are shown for (A) the stimulus effect (rich/lean) on accuracy, (B) the stimulus effect on RT, (C) the response effect (“rich”/ “lean”) on accuracy, (D) the response effect on RT, (E) response bias, and (F) discriminability.

**Figure S5**

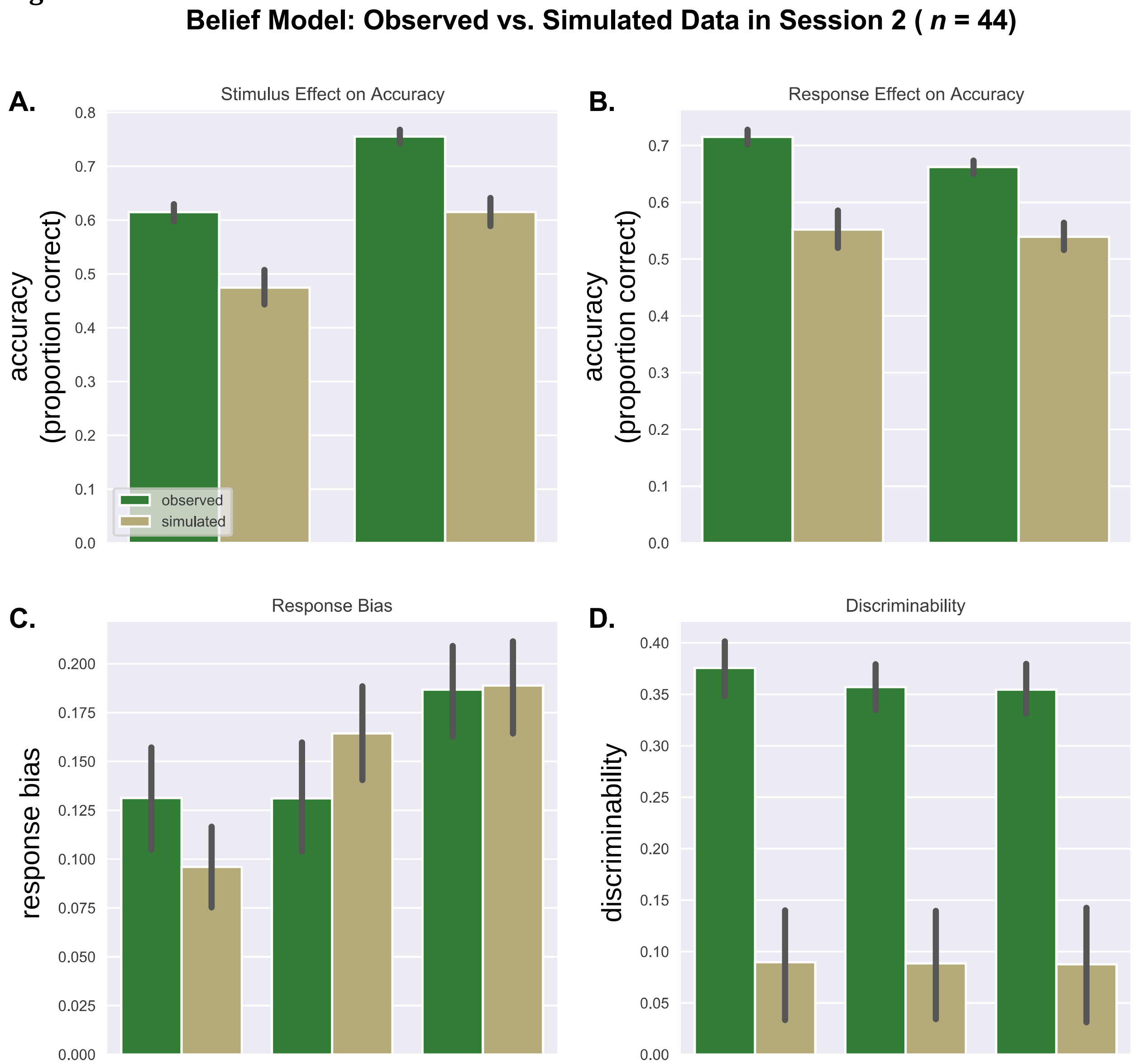


*Note*. Observed data from Session 2 vs. data from 500 simulations, generated using the belief model. Results are shown for (A) the stimulus effect (rich/lean) on accuracy, (B) the response effect ("rich"/ "lean") on accuracy, (C) response bias, and (D) discriminability.

**Figure S6**

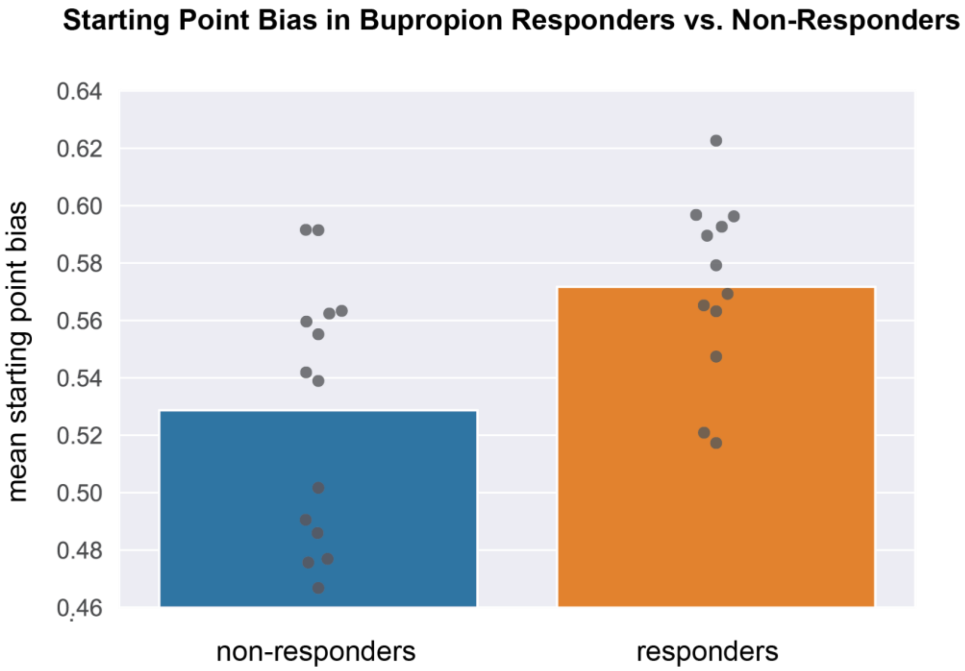


*Note.* Mean starting point bias estimates for bupropion responders vs. non-responders, given non-response to placebo.

**Table S1**

*Demographic Data*

| Session | *N* | Age | Number Females | Years of Education | Number White | Number Hispanic or Latino |
|---|---|---|---|---|---|---|
| 1 | 49 | 29±10 | 25 (51%) | 15±2 | 35 (71%) | 8 (16%) |
| 2 | 44 | 28±9 | 20 (45%) | 15±2 | 34 (77%) | 6 (14%) |

*Note*. Mean±SD values are given for age and years of education. Session 1 data were collected at baseline, before randomization to placebo or bupropion; Session 2 data were collected approximately three weeks later (mean±S.D. = 24±5 days between sessions). Age data were missing for one participant in Sessions 1 and 2.

**Table S2**

*Mean (SD) Response Bias, Discriminability, and Reward Totals by Session and Block*

| | Response bias | Discriminability | Reward total |
|---|---|---|---|
| **Session 1 (*n* = 49)** | | | |
| Block 1 | 0.09 (0.18) | 0.37 (0.19) | 38.0 (2.39) |
| Block 2 | 0.14 (0.18) | 0.38 (0.18) | 38.6 (2.06) |
| Block 3 | 0.16 (0.16) | 0.43 (0.19) | 39.0 (1.95) |
| **Session 2 (*n* = 44)** | | | |
| Block 1 | 0.13 (0.18) | 0.37 (0.18) | 38.4 (2.15) |
| Block 2 | 0.13 (0.19) | 0.36 (0.15) | 38.9 (2.15) |
| Block 3 | 0.19 (0.16) | 0.35 (0.17) | 38.6 (2.18) |

**Table S3**

*Results of Regressing Response Bias and Discriminability on HDDM Parameters*

| Parameter | *B* [95% CI] | *SE* | β | *t*-value | *p*-value |
|---|---|---|---|---|---|
| **Response Bias: Session 1** | | | | | |
| Threshold (*a*) | 0.01 [-0.14,0.16] | 0.08 | 0.02 | 0.12 | 0.904 |
| Non-decision time (*t*) | -0.16 [-0.73,0.41] | 0.28 | -0.07 | -0.55 | 0.583 |
| Drift rate (*v*) | -0.03 [-0.14,0.08] | 0.05 | -0.07 | -0.49 | 0.626 |
| Starting bias (*z*) | 1.86 [1.17,2.56] | 0.35 | 0.64 | 5.38 | < 0.001 |
| **Discriminability: Session 1** | | | | | |
| Threshold (*a*) | 0.29 [0.23,0.35] | 0.03 | 0.39 | 9.85 | < 0.001 |
| Non-decision time (*t*) | 0.12 [-0.10,0.34] | 0.11 | 0.04 | 1.07 | 0.289 |
| Drift rate (*v*) | 0.49 [0.45,0.53] | 0.02 | 1.02 | 23.13 | < 0.001 |
| Starting bias (*z*) | -0.25 [-0.52,0.03] | 0.14 | -0.07 | -1.80 | 0.078 |
| **Response Bias: Session 2** | | | | | |
| Threshold (*a*) | 0.01 [-0.17,0.19] | 0.09 | 0.02 | 0.10 | 0.922 |
| Non-decision time (*t*) | -0.25 [-0.76,0.26] | 0.25 | -0.13 | -0.98 | 0.332 |
| Drift rate (*v*) | -0.00 [-0.13,0.13] | 0.06 | -0.01 | -0.05 | 0.962 |
| Starting bias (*z*) | 2.58 [1.50,3.66] | 0.53 | 0.62 | 4.87 | < 0.001 |
| **Discriminability: Session 2** | | | | | |
| Threshold (*a*) | 0.25 [0.18,0.32] | 0.03 | 0.42 | 7.25 | < 0.001 |
| Non-decision time (*t*) | -0.05 [-0.24,0.14] | 0.10 | -0.02 | -0.51 | 0.611 |
| Drift rate (*v*) | 0.49 [0.44,0.54] | 0.02 | 1.17 | 20.16 | < 0.001 |
| Starting bias (*z*) | 0.13 [-0.28,0.54] | 0.20 | 0.03 | 0.64 | 0.523 |

**Table S4**

*Retest Reliability of the HDDM and Belief Models*

| Parameter | Pearson *r*-value | *p*-value |
|---|---|---|
| **HDDM** | | |
| Threshold (*a*) | 0.67 | < 0.001 |
| Non-decision time (*t*) | 0.36 | 0.036 |
| Drift rate (*v*) | 0.63 | < 0.001 |
| Starting bias (*z*) | 0.50 | 0.003 |
| **Belief Model** | | |
| Reward sensitivity | 0.06 | 0.727 |
| Instruction sensitivity | 0.33 | 0.054 |
| Learning rate | 0.25 | 0.148 |
| Belief | 0.27 | 0.123 |
| Initial bias | -0.46 | 0.006 |

*Note*. These analyses were run on 34 participants with usable PRT data at Sessions 1 and 2, regardless of assignment to placebo or bupropion.

**Table S5**

*Results of Regressing Response Bias and Discriminability on Belief Model Parameters*

| Parameter | *B* [95% CI] | *SE* | β | *t*-value | *p*-value |
|---|---|---|---|---|---|
| **Response Bias: Session 1** | | | | | |
| Reward sensitivity | 0.15 [0.06,0.24] | 0.04 | 0.53 | 3.30 | 0.002 |
| Instruction sensitivity | -0.13 [-0.26,-0.00] | 0.06 | -0.28 | -2.02 | 0.049 |
| Learning rate | 0.04 [0.02,0.06] | 0.01 | 0.63 | 4.05 | < 0.001 |
| Belief | 0.16 [0.03,0.29] | 0.06 | 0.36 | 2.52 | 0.016 |
| Initial bias | -0.25 [-0.86,0.36] | 0.30 | -0.10 | -0.82 | 0.415 |
| **Discriminability: Session 1** | | | | | |
| Reward sensitivity | -0.01 [-0.04,0.03] | 0.02 | -0.02 | -0.35 | 0.729 |
| Instruction sensitivity | 0.38 [0.34,0.43] | 0.02 | 0.66 | 16.28 | < 0.001 |
| Learning rate | -0.01 [-0.01,0.00] | 0.00 | -0.08 | -1.83 | 0.075 |
| Belief | 0.26 [0.21,0.31] | 0.02 | 0.46 | 10.91 | < 0.001 |
| Initial bias | -0.08 [-0.30,0.15] | 0.11 | -0.03 | -0.68 | 0.499 |
| **Response Bias: Session 2** | | | | | |
| Reward sensitivity | 0.22 [0.13,0.31] | 0.04 | 0.67 | 4.89 | < 0.001 |
| Instruction sensitivity | -0.00 [-0.12,0.11] | 0.06 | -0.01 | -0.05 | 0.962 |
| Learning rate | 0.05 [0.03,0.07] | 0.01 | 0.71 | 5.21 | < 0.001 |
| Belief | 0.06 [-0.08,0.19] | 0.07 | 0.10 | 0.82 | 0.420 |
| Initial bias | -0.29 [-0.90,0.31] | 0.30 | -0.11 | -0.99 | 0.329 |
| **Discriminability: Session 2** | | | | | |
| Reward sensitivity | -0.04 [-0.09,0.00] | 0.02 | -0.12 | -1.88 | 0.068 |
| Instruction sensitivity | 0.41 [0.35,0.47] | 0.03 | 0.80 | 14.02 | < 0.001 |
| Learning rate | -0.01 [-0.02,-0.00] | 0.00 | -0.14 | -2.13 | 0.040 |
| Belief | 0.20 [0.13,0.27] | 0.03 | 0.32 | 5.67 | < 0.001 |
| Initial bias | 0.25 [-0.06,0.57] | 0.15 | 0.09 | 1.65 | 0.107 |
